# Electrochemical Corey–Winter reaction. Reduction of thiocarbonates in aqueous methanol media and application to the synthesis of a naturally occurring α-pyrone

**DOI:** 10.3762/bjoc.14.41

**Published:** 2018-03-02

**Authors:** Ernesto Emmanuel López-López, José Alvano Pérez-Bautista, Fernando Sartillo-Piscil, Bernardo A Frontana-Uribe

**Affiliations:** 1 Centro Conjunto de Investigaciones en Química Sustentable UAEMéx-UNAM, Km 14.5 Carretera Toluca Atlacomulco San Cayetano-Toluca, 50200 Estado de México, México; 2 Centro de Investigación de la Facultad de Ciencias Químicas, Benemérita Universidad Autónoma de Puebla (BUAP), 14 Sur Esq. San Claudio, Col. San Manuel, 72570 Puebla, México; 3 Instituto de Química, Universidad Nacional Autónoma de México, Circuito exterior, Ciudad Universitaria, 04510 Ciudad de México, Mexico

**Keywords:** Corey–Winter reaction, electrosynthesis, 6-pentyl-2*H*-pyran-2-ones, reduction, thiocarbonates

## Abstract

An electrochemical version of the Corey–Winter reaction was developed giving excellent results in aqueous methanol media (MeOH/H_2_O (80:20) with AcOH/AcONa buffer 0.5 M as supporting electrolyte), using a reticulated vitreous carbon as cathode in a divided cell. The electrochemical version is much more environmentally friendly than the classical reaction, where a large excess of trialkyl phosphite as reducing agent and high temperatures are required. Thus, cathodic reduction at room temperature of two cyclic thiocarbonates (−1.2 to −1.4 V vs Ag/AgCl) afforded the corresponding alkenes, *trans*-6-(pent-1-enyl)-α-pyrone and *trans*-6-(pent-1,4-dienyl)-α-pyrone, which are naturally occurring metabolites isolated from *Trichoderma viride* and *Penicillium*, in high chemical yield and with excellent stereo selectivity.

## Findings

**Scheme 1:**
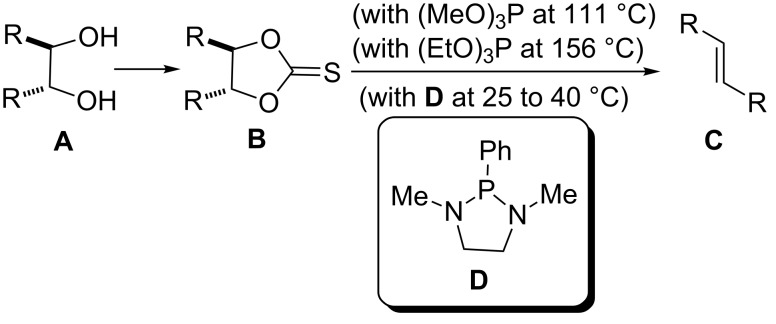
The Corey–Winter reaction in general.

The Corey–Winter reaction (also known as the Corey–Winter reductive olefination) is a chemical transformation that permits the conversion of 1,2-diols **A** into *E*-alkenes **C** via the formation and reduction of a cyclic thiocarbonate intermediate **B** (Scheme 1) [1–2]. In general this reaction provides moderate to good yields, but the use of large quantities of toxic and dangerous phosphorylated reductive reagents (e.g., trimethylphosphite employed as both reagent and solvent in the reaction) and the necessity of high temperatures, makes this synthetic protocol inappropriate for the stereospecific preparation of olefins in large scale [3]. Furthermore, the introduction of 1,3-dimethyl-2-phenyl-1,3,2-diazaphospholidine (**D**, Corey–Hopkins reagent) as the reductive reagent, has resulted in the development of a milder Corey–Winter protocol, which can be now carried out near to room temperature (Scheme 1) [4]. Despite this advantage, the low availability and high cost of this reagent [5] makes this reaction difficult to be used in industry.

**Scheme 2:**
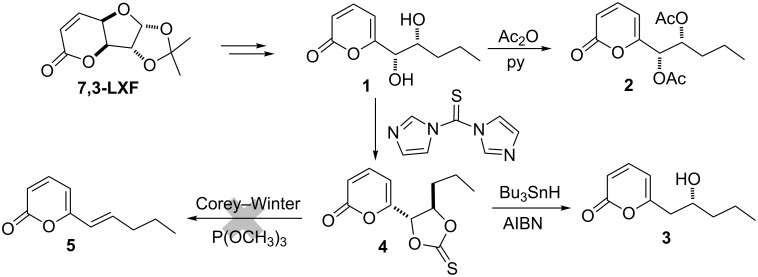
Proposed route for the synthesis of metabolites isolated from *Trichoderma* and *Penicillium* species from **7,3-LXF**.

Previously, starting from the versatile chiron 7,3-lactone-xylofuranose (**7,3-LXF**) [6], the first non-biological synthesis of chiral 6-pentyl-2*H*-pyran-2-ones **1**–**3** was reported by our research group [7]. These molecules proved to be enantiomers of metabolites isolated from *Trichoderma spp* and *Penicillium isolates*. Unfortunately, our efforts for obtaining the natural metabolite *trans*-6-(pent-1-enyl)-α-pyrone (**5**) (isolable from *Trichoderma viride* [8]) via a Corey–Winter reaction with the cyclic thiocarbonate **4** were unsuccessful (Scheme 2).

Electrochemical reactions are very useful in organic synthesis [9–14]. They possess unique features including the ability to control the redox power [15], the substitution of large scale toxic or dangerous conventional redox reagents by sustainable and inexpensive electrical current [16–19], the ease with which umpolung reactivity is achieved [20–22], the access to unstable intermediates of great utility in organic synthesis [23–25], and in most of the cases, the use of mild reaction conditions. Importantly, they also represent a useful way to fulfilling important aspects of the green chemistry [26–28], making the synthetic pathways safer and more economical. Therefore, electrosynthetic reactions can be advantageous not only for solving synthetic problems where a redox step fails with a classical redox reagent, but also because the use of toxic-dangerous reagents is avoided [15,29–30]. Thus, a combination of the classical chemical approach with the electrochemical in a synthetic route can result in an improved final yield diminishing chemical waste [15,31].

Having in mind that the Corey–Winter reaction is in fact a reductive chemical process between the thiocarbonate moiety and the phosphorylated reagent, which oxidize P(III) to P(V), we anticipated that a cathodic reduction process applied to the same cyclic thiocarbonate **4** would provide the desired target molecule **5**. In this letter we report the electrochemical behavior of thiocarbonates **4** and **6** in aqueous methanol MeOH/H_2_O (80:20) with 0.5 M AcOH/AcONa buffer as supporting electrolyte, as well as the results of several reductive electrolysis performed under green chemistry conditions.

**Scheme 3:**
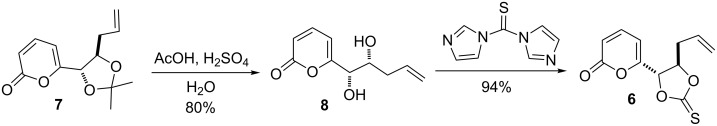
Preparation of thiocarbonate precursor **6** from pyrone dioxolane **7**.

Cyclic thiocarbonate **6** was prepared in two steps from pyrone dioxolane **7** [7]. Acid hydrolysis of **7** to 1,2-diol **8** followed by the reaction with 1,1’-thiocarbonyldiimidazole afforded thiocarbonate precursor **6** in high overall yield (Scheme 3). Compounds **6** and **8** were prepared in a similar manner as described in reference [7].

**Figure 1:**
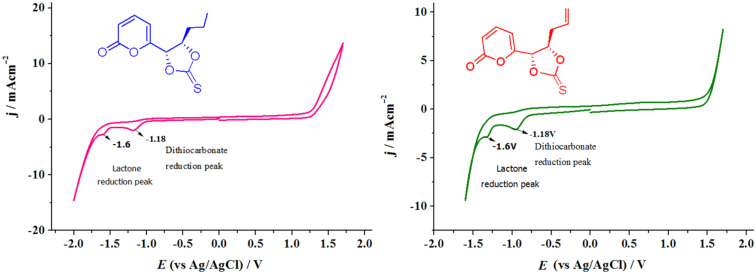
Cyclic voltammetry of thiocarbonates **4** (left) and **6** (right); *c* **=** 1 × 10^−3^ M, N_2_ bubbling 5 min, WE = vitreous carbon, CE = Pt wire, RE = Ag/AgCl in MeO/H_2_O (80:20) in AcOH/AcONa buffer 0.5 M media.

With thiocarbonates **4** and **6** in our hands, their electrochemical behavior was studied in the MeOH/H_2_O media previously described (Figure 1). This mixture of solvents has been used for the electrochemical preparation of anilines, hydroxylamines and nitroso compounds via the reduction of aromatic nitro derivatives [32–33], showing a convenient electrochemical window with graphitic electrodes (−1.75 to 1.25 V vs Ag/AgCl) and giving good environmental compatibility. Cyclic voltammetry of thiocarbonates **4** and **6** using a vitreous carbon electrode, showed two irreversible reduction peaks located at −1.18 and −1.6 V, respectively (Figure 1), indicating the possibility of reducing these compounds in two electrochemical steps under the selected conditions.

In order to determine which functional group is reduced, an electrolysis using 0.25 mmol of compound **4** was carried out under controlled potential after the first reduction peak observed in cyclic voltammetry (−1.45V vs Ag/AgCl) in a divided (sintered glass) H-type cell fitted with a reticulated vitreous carbon cathode and a stainless steel anode (see Supporting Information File 1 for details). When 2.2 F/mol were consumed (ca. 1.5 h), TLC control of the electrolytic solution showed the total consumption of the starting material and the appearance of a less polar compound without other secondary compounds.

**Scheme 4:**
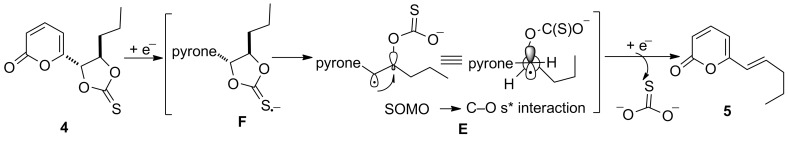
Putative reaction mechanism of the electrochemical Corey–Winter reaction.

After reaction work-up, ^1^H and ^13^C NMR spectra of the extracted product were in full agreement with compound **5**, which was previously isolated from *Trichoderma viride* [8]. This fact let us to conclude that the thiocarbonate group was reduced selectively to the *trans*-alkene in a Corey–Winter-type reaction [2–3] giving the targeted structure **5** in 95% isolated yield (Scheme 4, Table 1, entry 1). The reaction occurred stereoselectively with no trace of *cis*-alkene isomer. The use of graphite electrodes instead of the reticulated vitreous carbon eroded the chemical yield to 80% (Table 1, entry 2). Cyclic voltammetry analysis of **4** showed a second reduction peak at −1.6 V (Figure 1), a value that agrees with that for reduction of the α,β-unsaturated ester functionality [34], which was not reduced under the reaction conditions.

**Table 1:** Electroreduction of thiocarbonates **4** and **6** in MeO/H_2_O 80:20 with AcOH/AcONa buffer 0.5 M as electrolytic media.^a^

entry	thiocarbonate	conditions	product and yield %

1	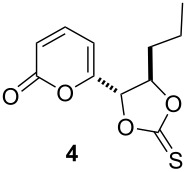 0.25 mmol	potentiostatic electrolysis −1.45 V vs Ag/AgCl, 2.2 F/mol RVC cathode, 1,5 h	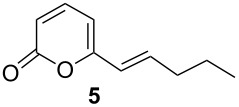 95–97^b^ current efficiency: 90%
2	0.25 mmol	potentiostatic electrolysis −1.45 V vs Ag/AgCl, 2.2 F/mol graphite plate cathode, 1,5 h	80 current efficiency: 90%
3	0.25 mmol	galvanostatic electrolysis *I* = 15–25 mA^c^ *j* = 7.5–12.5 mA/cm^2 d^ 2.5 F/mol, RVC cathode	95 current efficiency: 80%
4	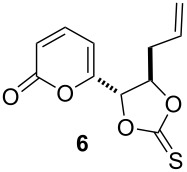 0.25 mmol	potentiostatic electrolysis −1.2 vs Ag/AgCl, 2.2 F/mol RVC cathode, 1,5 h	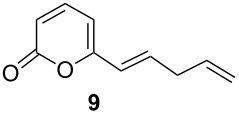 94–97^b^ current efficiency: 90%
5	0.25 mmol	galvanostatic electrolysis *I* = 15–25 mA^c^ *j* = 7.5–12.5 mA/cm^2 d^ 2.5 F/mol, RVC cathode	93 current efficiency: 80%

^a^All the experiments were carried out with stainless steel as anode. The potentiostatic and galvanostatic experiments were carried out in a H-cell divided cell with 25 mL of electrolytic media per compartment. ^b^Two reactions under the same conditions were carried out. ^c^Lowest and highest current used for an efficient reaction; when the lowest is used the reaction takes 1 h to consume the starting material and with the highest 30 min are required. ^d^A geometrical area of 2 cm^2^ was used to calculate *j*; see Supporting Information File 1 for calculation details.

The high stereospecificity of this electrochemical Corey–Winter-type reaction might be explained in terms of the stabilizing β-oxygen effect [35–36] of the radical intermediate **E**. This stereoelectronic interaction, which refers to the beneficial effect of β-oxygen substituents in radical deoxygenation [37], is dramatically favoured when the radical precursor group (or atom) is oriented antiperiplanar to the C−O bond via orbital interaction between the SOMO with the C−O σ* orbital [35]. Therefore, stabilization of intermediate **E**, which is formed by β-fragmentation of radical anion **F**, is maximum when is locked in an antiperiplanar conformation (Scheme 4). Therefore, this electrochemical reaction represents a promising way to produce *trans*-olefins from their respective thiocarbonates, overcoming thus the toxicity and safety issues of the classical Corey–Winter reaction.

When potential-controlled electrolysis (PCE) is used a particular functional group in a molecule, in this case the thiocarbonate vs the α,β-unsaturated ester, can be selectively reduced. Under PCE feasibility and affordability of the reaction are limited by the potentiostat, because this device is rarely available in an organic chemistry laboratory and it can be expensive to acquire it. In the other hand, current controlled electrolysis (CCE), where the electrode potential control is made indirectly by the experimental conditions, requires an ordinary and easy available direct current power source, therefore, reduction of compound **4** was attempted under CCE conditions. After a series of experiments, compound **5** was satisfactorily obtained using 25 mA (*j* = 12.5 mA/cm^2^) with 95% yield using reticulated vitreous carbon in 30 min reaction in the aqueous methanol medium (Table 1, entry 3). The current efficiency decreases a little (80%) but remains in acceptable values for an attractive electrochemical reaction. This is due to the lack of potential control and when the concentration of the starting material decreases the electrode potential shifts to more negative values. The same potential controlled electrolysis protocol used for **4** was applied to the alkene thiocarbonate derivative **6**, but reducing it at −1.2 V vs Ag/AgCl, and likewise, quantitative chemical yield of the corresponding *E*-alkene **9** was obtained (Table 1, entry 4). Also, the CCE of **6**, using the same current density previously optimized for compound **4**, gave **9** as the sole product in high yield (Table 1, entry 5). The reaction proceeds also with 15 mA (*j* = 7.5 mA/cm^2^); however, the electrolysis occurs slowly (1 h), whereas at 25 mA (*j* = 12.5 mA/cm^2^) the reaction is completed in 30 min; in both cases very good yields were obtained. When higher current density values for the electrolysis were used, TLC showed the formation of several byproducts. This indicates that the second reduction peak observed in cyclic voltammetry associated with the unsaturated ester function was reached.

There are several aspects of the reaction that are currently under study to generate a robust and general olefination electrochemical method via the thiocarbonate reduction; nevertheless, this letter shows for the first time the potential use of electrochemistry with this functional group using green reduction conditions. Application of this methodology to the synthesis of other biologically important products, the study of the reaction scope because other thiocarbonate derivatives seem to be not very reactive, as well as mechanistic details are under investigation and will be reported soon.

## Conclusion

A new approach to convert thiocarbonates derived from 1,2-diols containing the 6-pentyl-2*H*-pyran-2-one framework to *trans*-alkenes by means of electrochemical reduction in an H-type separated cell was developed. The thiocarbonate functional group can be reduced using a vitreous carbon electrode in MeOH/H_2_O 80:20 with AcOH/AcONa buffer 0.5 M as electrolytic media. We term this transformation as the “Electrochemical Corey–Winter (ECW) reaction”. This new environmentally friendly process was used to synthetize a metabolite isolated from *Trichoderma viride* in high yield. Alkenes and α,β-unsaturated ester functionalities are stable under the electrolysis conditions. The reaction can be driven by both, controlled potential and constant current electrolysis with excellent results, which is convenient for application in organic chemistry laboratories.

## Supporting Information

File 1 Experimental procedures and analytical data.
