# Supplementary material for: Electrochemical Corey–Winter reaction. Reduction of thiocarbonates in aqueous methanol media and application to the synthesis of a naturally occurring α-pyrone
Source: Beilstein J Org Chem. 2018 Mar 2;14:547–52. doi: 10.3762/bjoc.14.41 (PMC5852465; doi:10.3762/bjoc.14.41)
Supplement: File 1 — Experimental procedures and analytical data. [file Beilstein_J_Org_Chem-14-547-s001.pdf]

Supporting Information
for

Electrochemical Corey–Winter reaction. Reduction of thiocarbonates in aqueous methanol media and application to the synthesis of a naturally occurring α-pyrone

Ernesto Emmanuel López-López[1], José Alvano Pérez-Bautista[2], Fernando Sartillo-Piscil[*2] and Bernardo A. Frontana-Uribe[*,1,3]

Address: [1]Centro Conjunto de Investigaciones en Química Sustentable UAEMéx-UNAM, Km 14.5 Carretera Toluca Atlacomulco San Cayetano-Toluca, 50200, Estado de México, México, [2]Centro de Investigación de la Facultad de Ciencias Químicas, Benemérita Universidad Autónoma de Puebla (BUAP). 14 Sur Esq. San Claudio, Col. San Manuel. 72570, Puebla, México and [3]Instituto de Química Universidad Nacional Autónoma de México, Circuito exterior Ciudad Universitaria, 04510 Ciudad de México, Mexico

Email: Bernardo A. Frontana-Uribe - bafrontu@unam.mx ; ORCID: 0000-0003-3796-5933; Fernando Sartillo-Piscil - fernando.sartillo@correo.buap.mx ; ORCID: 0000-0002-4322-7534

*Corresponding author

Experimental procedures and analytical data

General information.

Commercially available reagents were used without further purification. Column chromatography (CC) was performed using silica gel (230–400 mesh) with solvents indicated in the text. Melting points were determined in an open capillary tube and are uncorrected. Optical rotations were measured in a digital polarimeter using the sodium D line (589 nm) and are reported as degrees at 20 °C. Concentrations are given as g/100 mL. Unless otherwise stated, ^{1}H NMR and ^{13}C NMR spectra were obtained on a 500 MHz

equipment. All samples were analyzed in $CDCl_3$ with TMS as internal reference using a relative scale in parts per million (ppm) for the chemical shift (δ) and Hz for the coupling constants (*J*). Splitting patterns are designated as follow: s, singlet; d, doublet; t, triplet; q, quartet; qu, quintet; m, multiplet; and br, broad. High resolution mass spectra (HRMS) were acquired in the electron-impact (EI) mode using a TOF mass analyzer or in fast-atom-bombardment (FAB) mode using a QMS mass analyzer.

Working electrode for electrolysis: Reticulated vitreous carbon electrode was made with 1 cm × 3 cm x 0.5 cm piece of sponge RVC (35 ppi ERG Materials Inc.) attached to a copper wire with an epoxy silver resin. The conductive epoxy was cured in the oven for 2 hours at 110 °C. The reticulated vitreous carbon area used in the experiments can be obtained from the specifications provided by the producer (ERG). This plot relates the area with the geometrical volume (ft^2/ft^3) for each porosity (pores per linear inch, ppi) available; 35 ppi have an area of 650 ft^2/ft^3 = 21.3 cm^2/cm^3. During the experiments the electrode was submerged 2 cm thus a volume of 1 cm^3 of the RVC electrode was exposed into the solution, which correspond to a submerged area of 21.3 cm^2. Because in porous electrodes the real electroactive area depends on the thickness and this value was not determined, geometrical current density is reported using the geometrical electroactive area (2 cm^2) of the face submerged to the anode. Please see the following link for details:

http://ergaerospace.com/technical-data/surface-area-of-duocel-foam/

Experimental procedure for the synthesis of 6-((1*S*,2*R*)-1,2-dihydroxypent-4-en-1-yl)-2*H*-pyran-2-one (8).

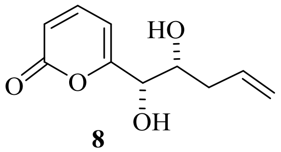

8

Pyrone dioxolane **7** (300 mg, 1.27 mmol) is dissolved in a mixture of $AcOH/H_2SO_4/H_2O$ (46:16:38) at room temperature. The resulting reaction mixture is stirred for 10 minutes or until consumption of the substrate (monitored by TLC). The reaction mixture is neutralized with a saturated aqueous solution of $NaHCO_3$ and extracted with ethyl acetate (3 × 20 mL). The organic phase is dried over anhydrous Na_2SO_4 and filtered through a pad of cotton. The filtrate was concentrated at reduced pressure and the residue was purified by column chromatography (SiO_2, ethyl acetate/hexane, 4/6) to afford 200 mg of **8** as white powder in 80% of yield. Mp = 91-93 °C. $[\alpha]_D^{20}$= –136.0 (*c* 1.0, CHCl3). ^{1}H NMR (500 MHz, $CDCl_3$, ppm) δ 2.41 (m, 2H), 2.51 (d, *J* = 4.5 Hz, 1H), 3.07 (d, *J* = 8.0 Hz, 1H), 4.10 (m, 1H), 4.33 (dd, *J* = 7.9, 2.9 Hz, 1H), 5.18 (apparent d, *J* = 1.0 Hz, 1H), 5.21 (m, 1H), 5.86 (m, 1H), 6.21 (d, *J* = 9.5 Hz, 1H), 6.40 (dt, *J* = 7.0, 1.0 Hz, 1H), 7.36 (dd, *J* = 9.4, 6.6 Hz, 1H). ^{13}C NMR (75 MHz, $CDCl_3$) δ 38.1, 70.8, 72.4, 102.8, 114.4, 119.1, 133.6, 143.7, 162.0, 164.9, HRMS (AIMS AccuTOFMS) *m/z* $[M+H]^+$ calcd for $C_{10}H_{12}O_4$: 197.0814; found: 197.0821.

Preparation of 6-((4*S*,5*R*)-5-allyl-2-thioxo-1,3-dioxolan-4-yl)-2*H*-pyran-2-one (6).

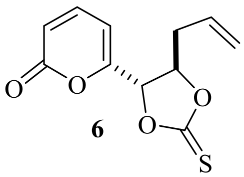

6

To a solution of **8** (41 mg, 0.2 mmol) in CH_2Cl_2 (5 mL) was added 1,1´-thiocarbonyldiimidazole (44.2 mg, 0.2 mmol). The resulting mixture was stirred 3 h at room temperature. The solvent was evaporated, and the residue was purified by column chromatography (SiO_2, ethyl acetate/hexane 7/3) to afford 48 mg of **6** as white powder in 94% of yield. Mp = 101–103 °C. $[\alpha]_D^{20}$= –216.7 (*c* 0.7, $CHCl_3$). ^1H NMR (500 MHz, $CDCl_3$, ppm) *δ:* 2.71 (m, 2H), 5.07 (q, *J* = 11.5, 5.5 Hz, 1H), 5.19 (d, *J* = 5.5, Hz, 1H), 5.32 (m, 1H), 5.35 (br. s, 1H), 5.78 (m, 1H), 6.36 (d, *J* = 9.5 Hz, 1H), 6.38 (d, *J* = 6.5 Hz, 1H), 7.36 (dd, *J* = 9.5, 6.5 Hz, 1H). ^{13}C NMR (75 MHz, $CDCl_3$) *δ* : 37.2, 80.6, 83.9, 104.0, 117.1, 121.9, 128.8, 142.4, 156.3, 159.8, 189.4. HRMS (AIMS AccuTOFMS) *m/z* $[M]^+$ calcd for $C_{11}H_{10}O_4S$: 238.0300; found: 238.0309

Electrochemical experiments

Cyclic voltammetry.

Pyrone derivatives containing the thiocarbonate group were analyzed by cyclic voltammetry in a 3 electrodes electrochemical cell using a vitreous carbon disk electrode (0.25 cm^2) as working electrode, platinum wire as counter electrode and Ag/AgCl as reference electrode. The electrolysis media used consisted in ethanol/water 80:20 mixture with a buffer of sodium acetate and acetic acid 0.5 M pH = 4.7 as supporting electrolyte. The scan rate potential was 100 mV/s starting at 0 V Ag/AgCl in anodic direction and then turn back to cathodic potentials to end again in 0 V. The first reduction peak observed was selected to carry out the preparative scale electrolysis of both studied molecules.

General procedure for the potential-controlled electrolysis.

The same electrolytic media used in the electroanalytical experiments was used to carry out the electrolysis in a divided (Sintered glass pore 4) H type cell (20 mL per chamber) at room temperature. The Ag/AgCl (reference electrode) and the reticulated vitreous carbon electrode (see general information, working electrode) were fitted in the same cell chamber (cathodic); a plate of stainless steel with 1 × 3 cm (counter electrode) was fitted in the other chamber (anodic) (Figure S1). In the cathodic chamber the thiocarbonate derivative (0.25 mmol) was dissolved by magnetic stirring, the solution was deoxygenated with N_2 for 10 min. and the potentiostat was set to the selected electrolysis potential (−1.45 V for compound **4** and −1.2 V for compound **6**) and turned on, under a gentle N_2 flux. The reaction was followed by thin layer chromatography. After ca. 1.30 min the starting

material disappeared and a new spot above the starting material was the only product observed in TLC. The cathodic chamber solution was separated and the chamber was rinsed with MeOH. The organic solvent of these solutions was distilled under reduced pressure using a rotary evaporator, and to the aqueous solution remain, 25% bicarbonate solution was added until pH = 7. The product was extracted with dichloromethane (3 × 50 mL) and the organic extract was dried with sodium sulfate, filtered and the solvent evaporated in the rotary evaporator. The crude of the reaction sent to NMR analysis, to confirm his identity showing pure compound.

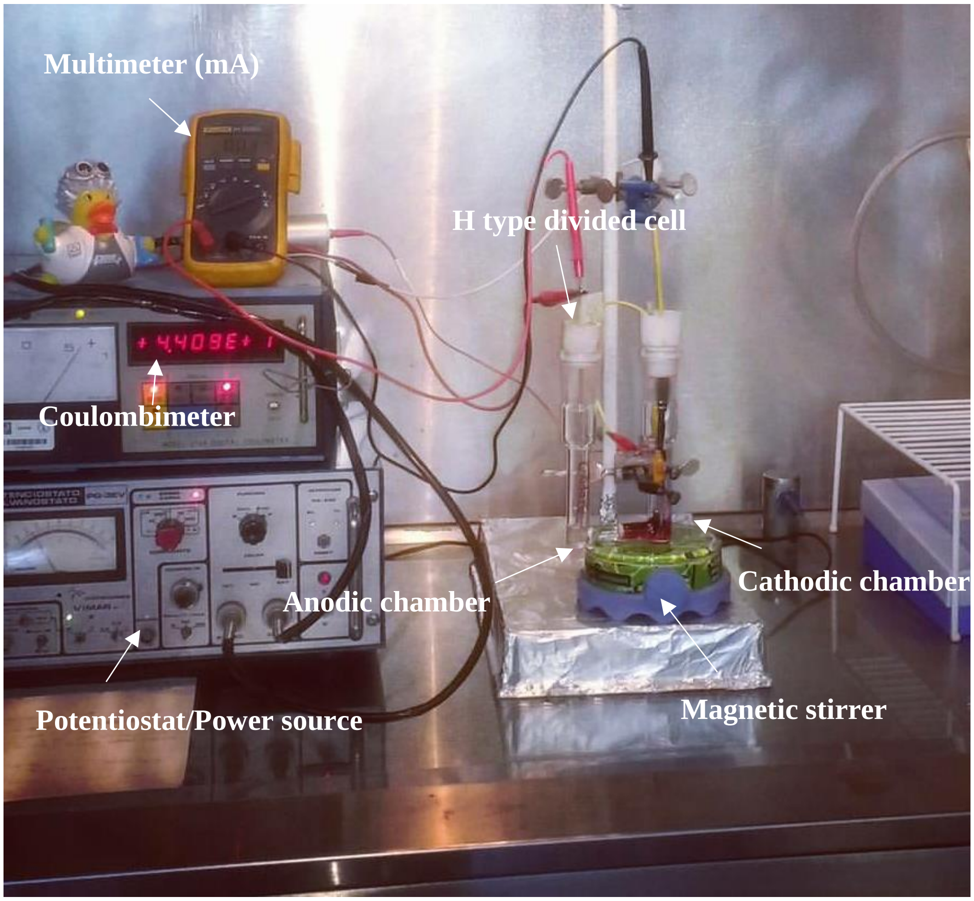

Figure S1. Electrolysis setup for the thiocarbonates reduction in hydroalcoholic media.

General procedure for the current-controlled electrolysis (CCE).

The same electrolytic media used in the electroanalytical experiments was used to carry out the electrolysis in a divided (Sintered glass pore 4) H type cell (20 mL per chamber) at room temperature. The reticulated vitreous carbon (see general information, working electrode) was fitted in the cathodic chamber; a plate of stainless steel with 1 x 3 cm

(counter electrode) was fitted in the anodic chamber (Figure S1). In the cathodic chamber the thiocarbonate derivative (0.25 mmol) was dissolved by magnetic stirring, the solution was deoxygenated with N_2 for 10 min. and the power source was set to the selected electrolysis current (j = 7.5–12.5 mA/cm^2) and turned on, under a gentle N_2 flux. Reaction was followed by thin layer chromatography, after ca. 30 min (12.5 mA/cm^2) the starting material disappeared and a new spot above the starting material was the only product observed in TLC. The cathodic chamber solution was separated and the chamber was rinsed with MeOH. The organic solvent of these solutions was distilled under reduced pressure using a rotary evaporator, and to the aqueous solution remain, 25% bicarbonate solution was added until pH = 7. The product was extracted with dichloromethane (3 × 50 mL) and the organic extract was dried with sodium sulfate, filtered and the solvent evaporated in the rotary evaporator. The crude of the reaction sent to NMR analysis, to confirm his identity showing pure compound.

trans-6-(pent-1-enyl)-α-pyrone (5)

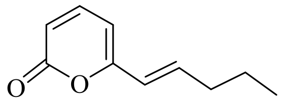

5

^{1}H NMR (300 MHz, Chloroform-*d*) δ 7.35 – 7.23 (m, 1H), 6.71 (dt, J = 15.1, 7.2 Hz, 1H), 6.16 (d, J = 9.3 Hz, 1H), 5.97 (d, J = 6.4 Hz, 1H), 2.21 (q, J = 7.3 Hz, 2H), 1.50 (q, J = 7.3 Hz, 2H), 0.94 (t, J = 7.4 Hz, 3H); ^{13}C NMR (75 MHz, $CDCl_3$) δ 162.05, 159.74, 143.84, 139.64, 121.66, 113.67, 103.02, 34.83, 21.84, 13.67. IR (ATR) cm^{-1}: 2959, 2930, 2872, 1719, 1649, 1535, 1776, 1095, 964, 790, 733, 550. (EI-MS) calcd. For $C_{10}H_{12}O_2$ 164.08 $(M+H)^+$ found m/z 164.09. HRMS (ESI TOF +) *m/z* $[M+H^+]^+$ calcd for $C_{10}H_{12}O_2$: 165.0910; found: 165.0908.

trans-6-(pent-1,4-dienyl)-α-pyrone (9)

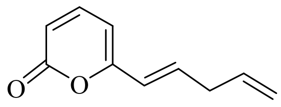

9

^{1}H NMR (300 MHz, Chloroform-*d*) δ 7.32 – 7.25 (m, 1H), 6.72 (s, 1H), 6.18 (d, J = 9.3 Hz, 1H), 5.99 (d, J = 6.1 Hz, 2H), 5.92 – 5.74 (m, 1H), 5.14 (d, J = 5.6 Hz, 1H), 5.09 (s, 1H), 2.98 (t, J = 6.5 Hz, 2H); ^{13}C NMR (75 MHz, $CDCl_3$) δ 161.96, 159.57, 143.82, 136.77, 134.63, 122.41, 117.06, 114.17, 103.53, 36.65. (EI-MS) calcd. For $C_{10}H_{10}O_2$ 162.07 $(M+H)^+$ found m/z 162.06._HRMS (ESI TOF +) *m/z* $[M+H^+]^+$ calcd for $C_{10}H_{10}O_2$: 163.0753; found: 163.0749.
